# Activation of Latent HIV Using Drug-Loaded Nanoparticles

**DOI:** 10.1371/journal.pone.0018270

**Published:** 2011-04-05

**Authors:** Michael Kovochich, Matthew D. Marsden, Jerome A. Zack

**Affiliations:** 1 Department of Microbiology, Immunology, and Molecular Genetics, University of California Los Angeles, Los Angeles, California, United States of America; 2 Division of Hematology and Oncology, Department of Medicine, David Geffen School of Medicine at University of California Los Angeles, Los Angeles, California, United States of America; Institut Pasteur Korea, Republic of Korea

## Abstract

Antiretroviral therapy is currently only capable of controlling HIV replication rather than completely eradicating virus from patients. This is due in part to the establishment of a latent virus reservoir in resting CD4+ T cells, which persists even in the presence of HAART. It is thought that forced activation of latently infected cells could induce virus production, allowing targeting of the cell by the immune response. A variety of molecules are able to stimulate HIV from latency. However no tested purging strategy has proven capable of eliminating the infection completely or preventing viral rebound if therapy is stopped. Hence novel latency activation approaches are required. Nanoparticles can offer several advantages over more traditional drug delivery methods, including improved drug solubility, stability, and the ability to simultaneously target multiple different molecules to particular cell or tissue types. Here we describe the development of a novel lipid nanoparticle with the protein kinase C activator bryostatin-2 incorporated (LNP-Bry). These particles can target and activate primary human CD4+ T-cells and stimulate latent virus production from human T-cell lines *in vitro* and from latently infected cells in a humanized mouse model *ex vivo*. This activation was synergistically enhanced by the HDAC inhibitor sodium butyrate. Furthermore, LNP-Bry can also be loaded with the protease inhibitor nelfinavir (LNP-Bry-Nel), producing a particle capable of both activating latent virus and inhibiting viral spread. Taken together these data demonstrate the ability of nanotechnological approaches to provide improved methods for activating latent HIV and provide key proof-of-principle experiments showing how novel delivery systems may enhance future HIV therapy.

## Introduction

Highly active antiretroviral therapy (HAART) can powerfully suppress HIV replication but does not clear virus from infected individuals. Various reservoirs of replication-competent HIV have been identified that may contribute to this persistence, the most prominent and well-characterized of these being latently infected CD4+ T-cells. Several attempts have been made to clear the latent HIV reservoir but these have not been successful in eliminating all latently infected cells or in preventing virus rebound upon cessation of therapy [1], [2], [3], [4]. Hence, complete eradication of HIV from infected individuals will probably require further improvements in latency activators and the development of a more optimized virus purging strategy.

One focus of latency research is the identification of factors that can activate HIV from latency and have the potential to be used in a clinical setting. Such factors have included histone deacetylase inhibitors, agonistic anti-CD3 antibodies, and cytokines such as interleukin (IL)-2 and IL-7 [2], [4], [5], [6], [7], [8]. A promising lead in this context is the protein kinase C (PKC) activator bryostatin, which is part of the marine macrolide class of molecules. Bryostatin has been demonstrated to activate latent HIV at nanomolar concentrations *in vitro* [9], [10], [11]. This molecule is believed to induce expression of HIV by activating NFκB [12], which is required for optimal transcription of viral mRNA from the HIV long terminal repeat promoter (LTR). Although bryostatin-1 has been tested in clinical trials for its anti-cancer properties [13], [14], [15], [16], [17], [18] there are no published studies using this class of compounds in clinical HIV purging strategies. Bryostatin-2 was used in our studies and has slightly different properties than the parent compound bryostatin-1, although both can activate PKC at nanomolar concentrations [13].

A second opportunity for improving latency activation is the development of enhanced delivery vehicles for existing molecules. Nanoparticle delivery systems possess several advantages over more traditional drug delivery methods. For example, nanoparticles can be used to modify drug solubility and bioavailability or to allow multiple drugs to be incorporated into the same delivery vehicle. Nanoparticles can also be targeted to particular cell or tissue types, providing specificity that may not normally be a characteristic of the incorporated drug. Numerous nanotechnological systems have been approved for clinical use, including over 20 products for the delivery of various classes of drugs [19], [20], [21], [22]. Liposomes represent one type of nanoparticle that has been a focus of basic research for over 40 years [23]. In the past a major drawback in the clinical use of liposomes was their rapid clearance due to uptake by mononuclear phagocytic cells. Recent improvements in liposomal formulations have reduced this uptake by coating the surface of particles with polymers such as polyethylene glycol, thereby increasing the blood circulation time [24]. These optimized liposomes have led to more clinically applicable delivery systems as evidenced by the over 10 FDA approved liposomal drug formulations, with several others in clinical trials [25]. One example of this is doxorubicin incorporated into liposomes, known as Doxil. In 1995 this became the first liposome based therapeutic to be approved by the FDA. Doxil was initially used for treating HIV-related Kaposi's sarcoma and was later approved for ovarian and other types of cancers. Advantages of Doxil over free drug properties included enhanced biodistribution and prolonged half life in blood circulation [26], [27]. Our particular study aimed to test the efficacy of a liposomal formulation of the HIV latency activator bryostatin-2 and to test whether this nanoparticle delivery platform can enhance the ability to activate latent HIV.

Our current work is a proof-of-concept study demonstrating that HIV latency activators can be packaged into nanoparticles, either alone or in conjunction with an antiretroviral drug. Bryostatin-2 loaded lipid nanoparticles (LNP-Bry) produced in this way have enhanced activity compared with drug alone, and can be further modified by adding targeting antibodies to make them more selective for CD4+ T cells. This is the first demonstration of the use of nanotechnology in HIV latency activation and may provide a direction for more effective therapy in future purging strategies.

## Results and Discussion

### LNP characterization and uptake in various cell types

It is becoming increasingly evident that HAART will not clear HIV infection from the body; thus patients must stay on therapy for the remainder of their lives. These therapies are associated with several toxicities; therefore, the field has begun to explore ways to purge residual viral reservoirs. One such approach involves activation of latent reservoirs, in the context of HAART, with the intent that the immune system will destroy the cells induced to produce virus. Our aim was to develop a lipid nanoparticle (LNP) delivery system that activates latent HIV and may have potential for purging viral reservoirs. Historically, nanoparticles have received particular attention in the area of cancer therapy; more recently, nanoparticle delivery systems have also been used in HIV antiretroviral research [28], [29], [30], [31], [32], [33]. However, these studies did not investigate the potential use of nanoparticles in the context of HIV latency.

**Figure 1:**
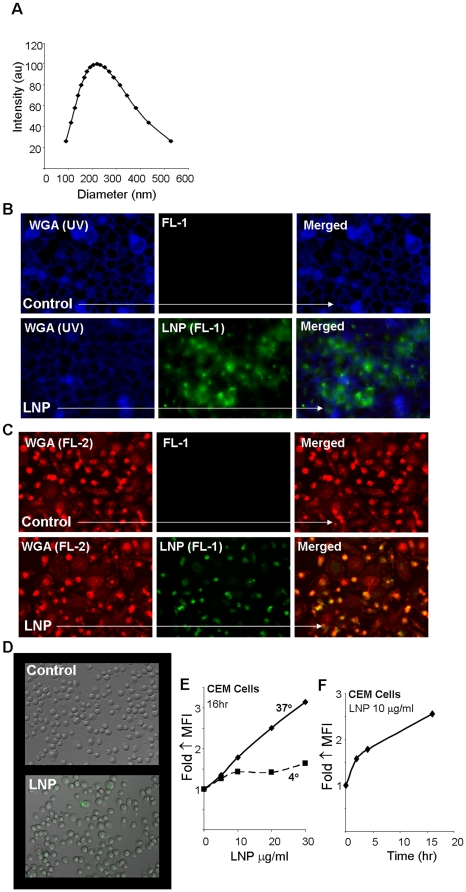
Lipid nanoparticle (LNP) characterization and uptake in various cell types. (A) LNP were synthesized and characterized for their size by dynamic light scattering. (B) The membrane stain wheat germ agglutinin (WGA) visualized in blue was used with fluorescent microscopy in order to visualize the uptake of LNP-FITC (green) after a 16 hr incubation with HeLa cells. (C) WGA visualized in red was used to observe LNP-FITC (green) uptake in primary macrophages. (D) CEM cells were visualized by phase contrast images with LNP (green) using fluorescent microscopy. (E) LNP uptake in CEM cells was dose and energy dependent as detected by fold increase in FITC mean fluorescent intensity (MFI) using flow cytometry. (F) LNP uptake in CEM cells also increased over time as detected by flow cytometry.

Bryostatin-2 was chosen for incorporation into the LNP due to the low effective dosage for latent HIV activation (1–10 nM) as well as the fact that this class of compounds have been widely used in clinical trials exploring its anti-cancer properties [13], [14], [15], [16], [17], [18]; therefore bryostatin preparations could possibly be more rapidly advanced to clinical trial for use in HIV therapy. Bryostatin-2 incorporation was efficient due to its small size (MW = 863 daltons) and hydrophobic nature, which allows it to be readily incorporated into the membrane of the LNP, forming nanoparticles with an average size of 219 nm (Fig. 1A). Further characterization showed efficient LNP uptake in various cell types (Fig. 1B–D). The punctate dots observed in HeLa cells and primary macrophages represent a classical sign of particles localized in cellular compartments such as caveolea pits or early endosomes. The diffuse layer of nanoparticles present in all three tested cell types more likely represents a fusion event where LNP are dispersing into the cellular membranes. LNP uptake in CEM T-cells was found to be efficient, and proved to be dose, temperature, and time dependent (Fig. 1E–F). The lipid nanoparticles were non-toxic in all cell types tested including peripheral blood mononuclear cells (PBMCs). These data demonstrate the ability of our LNP delivery system to be efficiently taken up into various cell types providing a platform in which we can effectively deliver our cargo of interest, including the HIV latency activator bryostatin-2 and the protease inhibitor nelfinavir.

### Effects of LNP-Bry on uninfected primary CD4+ T cells and latently infected cells

**Figure 2:**
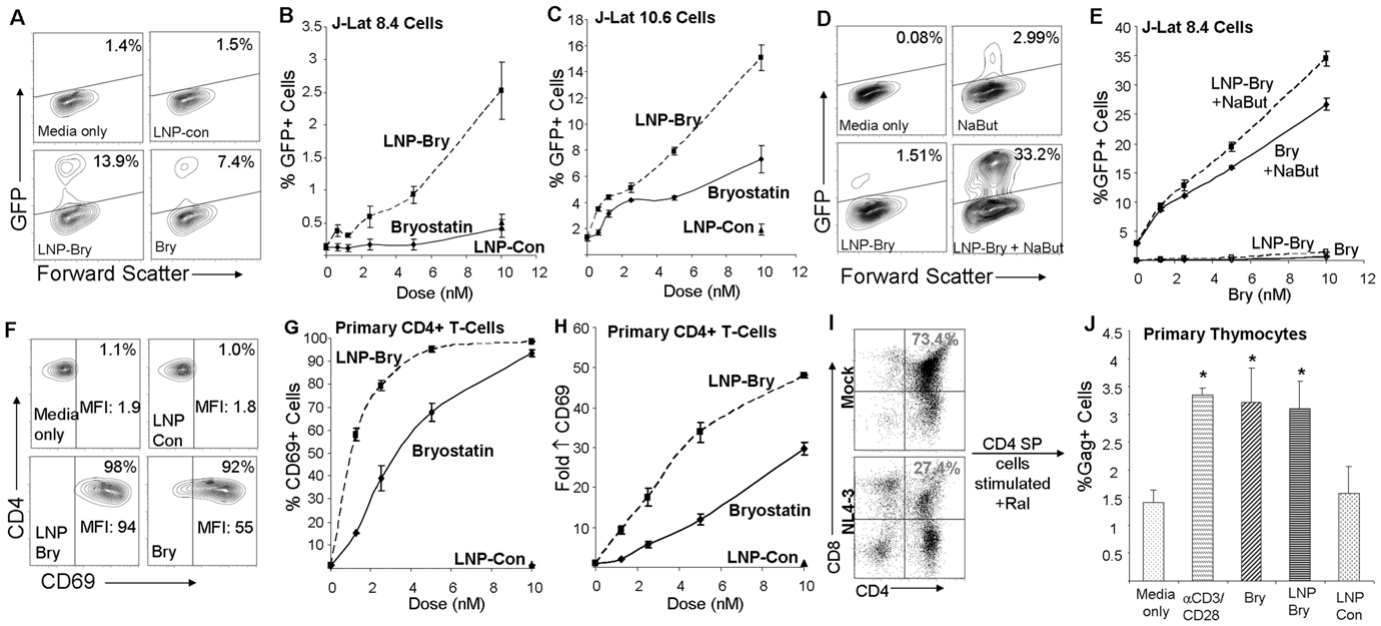
Effects of LNP-Bry on latent virus expression and induction of CD69. (A) Representative flow cytometry plots of GFP expressing J-Lat 10.6 cells incubated with 10 nM bryostatin-2 or brostain-2 loaded LNP (LNP-Bry). (B) Percentage GFP+ cells was measured after incubation of LNP-Bry or bryostatin-2 with J-Lat 8.4 cells for 16 hr. (C) Percentage GFP+ cells was measured after incubation of LNP-Bry or bryostatin with J-Lat 10.6 cell lines for 16 hr. (D) Representative flow cytometry plots displaying the synergistic effect of 2 mM sodium butyrate (NaBut) in conjunction with LNP-Bry in J-Lat 8.4 cells. (E) J-Lat 8.4 cells were incubated with a fixed dose of NaBut (2 mM) and increasing dose of bryostatin-2 or LNP-Bry. (F) Representative flow cytometry plots displaying CD69 induction in primary resting CD4+ cells after incubation with LNP-Bry or bryostatin-2 for 16 hr. (G) Percentage CD69+ cells as detected by flow cytometric analysis. (H) Fold increase in CD69 mean fluorescence intensity was measured after incubation with increasing amounts of LNP-Bry or bryostatin-2 in primary CD4+ cells. (I) Flow cytometry plots displaying the CD4+/CD8+ cell profiles from mock or NL4-3 infected SCID-hu (Thy/Liv) implants. (J) In the presence of 1 µM raltegravir, CD4 single-positive thymocytes were stimulated *ex-vivo* with anti-CD3/CD28 beads, 10 nM bryostatin-2 or 10 nM LNP-Bry for 48 hr and assayed for intracellular Gag protein by flow cytometry. Error bars indicate the standard deviation of triplicate data points and are representative of at least 2 experiments. * p<0.01 as compared with media only (infected non-stimulated cultures) or LNP-con (non-drug loaded nanoparticles) in a paired t-test.

To determine whether bryostatin-2 loaded nanoparticles (LNP-Bry) are capable of stimulating HIV from latency, we first utilized the J-Lat cell line model, which expresses GFP upon activation of latent HIV [34]. LNP-Bry were more effective than drug alone at activating latent HIV at all doses tested (Fig. 2A–C). This may be due to the increased drug solubility by the lipid nanoparticle or increased local concentration of the drug near the cellular membrane. Indeed, our uptake data indicate that LNP typically associate with the membranes in T-cells and this interaction may be responsible for the increased drug effects observed in our system. It is interesting to note that the site of action for bryostatin-2 is protein kinase C on the intracellular membrane, so it is possible that the LNP-Bry provides a more efficient means of delivering drug to this target. This is the first empirical evidence that incorporation of bryostatin-2 into nanoparticles may provide advantages over using drug alone. Consistent with the data presented here, previous studies of other LNP-drug preparations have also documented increased drug uptake in cells exposed to lipid-drug formulations compared with drug alone [30], [35].

Previous reports have described a synergistic HIV stimulatory effect between HDAC inhibitors and PKC activators such as prostratin [36] and bryostatin [10]. We found that the combination of LNP-Bry and the HDAC inhibitor sodium butyrate have a far superior effect than either latency activator alone (Fig. 2D,E). It is important to note that other HDAC inhibitors such as SAHA or VPA have also been shown to synergize with PKC activators; therefore these may also prove to be effective in combination with nanoparticle delivery of PKC activators [36]. Finally, since any purging strategy would probably necessitate effective stimulation in primary CD4+ T-cells, we tested the ability of LNP-Bry to induce the early activation marker CD69 (Fig. 2F–H). Similar to our previous data, LNP-Bry was more effective at inducing CD69 expression compared with bryostatin-2 alone. Again, this demonstrates the advantage of using this LNP system for the delivery of bryostatin-2.

In order to test the ability of LNP-Bry to activate latent virus in a primary cell model we utilized the SCID-hu (Thy/Liv) mouse model for HIV latency [7], [37], [38], [39], [40], [41], [42]. Briefly, this model involves transplantation of human fetal liver (as a source of stem cells) and human fetal thymus under the kidney capsule of immunodeficient mice. We have previously reported a high proportion of latently infected mature human thymocytes in this model [39]. The CD4/CD8 expression profile of mock and infected implants at 4 weeks post-infection was consistent with our previous data using this model, where CD4+CD8+ (double-positive) cells are preferentially depleted by HIV (Fig. 2I). Mature CD4 single-positive cells were then isolated and stimulated for 2 days in the presence of 1 µM raltegravir to prevent additional virus infection during activation. Anti-CD3/anti-CD28 beads served as a costimulation positive control. LNP-Bry and bryostatin-2 were each capable of stimulating latent virus as assessed by intracellular Gag protein expression (Fig. 2J). Culture supernatants were also assayed for HIV p24 Gag protein using ELISA, and activation from latency by LNP-Bry was again evident. Taken together these data provide a proof–of-concept that nanoparticle delivery of bryostatin-2 can improve the activation of latent HIV.

### Incorporation of both a protease inhibitor and latency activator into the same nanoparticle

**Figure 3:**
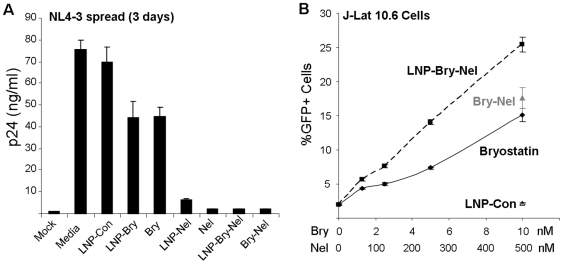
Simultaneous incorporation of the protease inhibitor nelfinavir (Nel) and bryostatin-2 (Bry) into the lipid nanoparticles (LNP-Bry-Nel) can both activate latent virus expression and inhibit viral spread. (A) CEM cells were infected with HIV_NL4-3_ and the cells were incubated for 3 days in the presence of various drug combinations including LNP-Bry-Nel. Viral p24 protein in the culture supernatant was measured by ELISA. (B) LNP-Bry-Nel was further tested for its ability to activate latent virus in J-Lat 10.6 cells as measured by induction of GFP expression. Error bars indicate the standard deviation of triplicate data points and are representative of at least 2 experiments. Media represents untreated infected cultures and LNP-con represents non-drug loaded nanoparticles.

One major potential benefit to the use of nanoparticles is the ability to incorporate multiple classes of drugs into the same delivery vehicle. Any HIV purging strategy would be performed in the presence of HAART, but it would be useful to incorporate an antiretroviral drug such as a protease inhibitor into the same particle that is used to activate latent virus to directly introduce higher levels of drug to the cells. Conceptually, this is similar to the idea of HAART intensification during reservoir purging, and it represents a safety feature whereby any virus induced by the nanoparticles to express would produce viral epitopes, but would be inactivated as it exits the host cell. This would allow subsequent targeting by the immune response in the absence of increased production of viable virus. This strategy may be particularly important if particles are developed to enter anatomical sites that are not easily penetrated by some antiretroviral drugs (such as the brain). To this end, we incorporated the protease inhibitor nelfinavir into our nanoparticles along with bryostatin-2 (LNP-Bry-Nel). This particle can effectively inhibit virus spread in a culture of CEM cells (Fig. 3A). The same particles were also able to stimulate latent HIV expression in J-Lat 10.6 cells (Fig. 3B). Hence, the LNP-Bry-Nel can effectively stimulate latent virus and also inhibit virus spread, which further exemplifies the potential benefits of using nanoparticles in HIV purging strategies.

### Targeting of LNP to primary CD4+ cells and delivery of bryostatin-2

**Figure 4:**
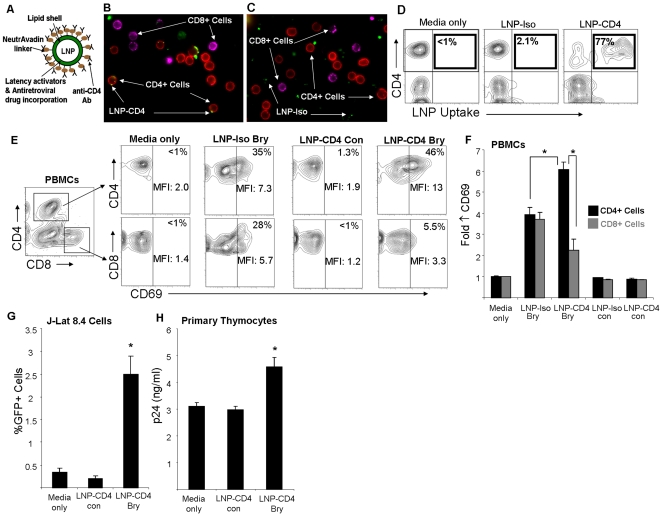
Targeting LNP to primary CD4+ cells and activation of latent virus expression. (A) Schematic representation showing construction of the LNP-CD4 (anti-CD4 coated nanoparticles) and LNP-Iso (isotype control antibody coated nanoparticles) with bryostatin-2 incorporation. (B) Fluorescent microscopy image displaying the targeting of LNP-CD4 (green) to CD4+ cells (red) while minimizing cellular association with CD8+ cells (violet) in the same culture of total peripheral blood mononuclear cells (PBMCs) after a 30 min exposure. (C) Fluorescent microscopy image displaying the lack of targeting by LNP-Iso (green) to CD4+ cells (red) or CD8+ cells (violet). (D) Quantification of LNP-CD4 uptake in PBMCs using flow cytometry (% cells CD4+ with increase in FITC-LNP uptake) after a 30 min exposure. (E) Representative flow cytometry plots of CD4+ and CD8+ cells analyzed for the induction of the early activation marker CD69 after incubation with CD4 targeting nanoparticles for 16 hr. (F) Bar graphs illustrating the fold increase in CD69 MFI in CD4+ and CD8+ cells after incubation with LNP-CD4 Bry. (G) LNP-CD4 Bry was further tested for its ability to stimulate latent virus expression in J-Lat 8.4 cells. (H) *Ex vivo* CD4 single-positive thymocytes isolated from infected SCID-hu (Thy/Liv) implants were incubated with LNP-CD4 Bry and viral p24 protein present in the culture supernatants was analyzed after 48 hr. Error bars indicate the standard deviation of triplicate data points and are representative of at least 2 experiments. * p<0.01 in a paired t-test as indicted in (F) and as compared with media only or LNP-con in (G) and (H).

Optimal therapeutics utilize the lowest possible effective dose while minimizing off-target effects. Nanoparticles can be modified to selectively target certain cells and thus optimize this balance of drug delivery to the target site versus non-targeted sites. In particular, liposomes offer a biodegradable vehicle which is capable of carrying a variety of substances to targeted sites [23] achieved either through passive or active targeting [19]. These targeting strategies may help prevent toxicity due to generalized immune activation during efforts to purge latent HIV. Therefore, we explored the possibility of adding a level of selectivity to latency activators that might reduce bystander cell activation. The most optimal HIV purging strategy would involve specific targeting of latently infected cells, but this may prove unachievable because there is no known distinguishing cell surface marker for a latently infected cell [42]. However, it may still be possible to activate only the subset of cells that may harbor the latent infection and in doing so minimize cellular activation of all non-targeted cells. Therefore for this proof-of-concept study we chose CD4 as our target receptor for possible latently infected cells. Targeted particles (LNP-CD4) did indeed target CD4+ cells in a PBMC culture (Fig. 4A–D) when compared with isotype control antibody coated particles (LNP-Iso). As expected LNP-Iso (non-targeting) showed no preference for activating CD4+ or CD8+ T-cells. In sharp contrast, when introduced into a mixed peripheral blood cell culture LNP-CD4 preferentially induced CD69 expression on CD4+ cells with minimal activation of CD8+ cells (Fig. 4E,F). LNP-CD4 was also capable of activating latent HIV in J-Lat 8.4 cells and primary thymocytes (Fig. 4G,H). These data demonstrate the ability of nanoparticles to target and activate latent HIV in CD4+ cells while minimizing cellular activation of non-targeted cells. It is important to note that when constructing the most optimal *in vivo* purging strategy, multiple types of nanoparticles that target different cells or tissue types could be used in combination. For example, while CD4 targeting nanoparticles may be useful in systemic T-cell targeting, unmodified liposomes have been documented to target lymphoid tissue due to their size and drainage into lymphatic vessels [43], [44], [45]. This type of combinatorial approach along with the idea that both latency activators and protease inhibitors will be delivered by the same vehicle may be used to enhance HIV latency purging strategies *in vivo*.

### Summary

In summary, our data illustrate the potential use of nanotechnology in HIV purging strategies. These data have provided a proof-of-concept study for the use of nanodelivery systems in HIV latency research. Taken together this study demonstrates several advantages that targeted drug loaded nanoparticles can provide in developing optimized strategies for HIV latency activation and will provide a foundation for future novel HIV purging strategies.

## Materials and Methods

### Lipid nanoparticle formation and characterization

All lipids were purchased from Avanti Polar Lipids. Liposomes were made by thin film evaporation methods described previously [46]. PC, PE, PS lipids were used at a 40∶40∶20 molar ratio. 2% FITC-PE was incorporated for fluorescently labeled lipids. Bryostatin-2 was purchased from LC Laboratories. The particles were purified from any free drug by PD 10 column (GE Healthcare) separation. LNP were then characterized for size using dynamic light scattering (Brookhaven Zeta Sizer Instrument). Immunoliposomes with biotinylated-PE lipid (0.1% incorporated) were engineered according to previous protocols [47]. Biotinylated LNP were coated with NeutrAvidin (NA) (Pierce) and purified from free protein using MW100 filters (Millipore). NA-coated LNP were then incubated with either anti-CD4 Ab or Isotype control Ab (1% w/w).

### Cellular uptake of LNP

HeLa cells [48] were cultured in Dulbecco's Modified Eagle Medium + penicillin/streptomycin (PS) + 10% fetal bovine serum (FBS). CD14+ monocytes were isolated using anti-CD14 magnetic beads (Miltenyi) and cultured for 1 week in macrophage media (Iscove's Modified Dulbecco's Medium + PS + 10% FBS + 5% human AB serum) containing 10 ng/ml M-CSF (macrophage colony stimulating factor). CEM cells [49] were cultured in RPMI + PS +10% FBS. Fluorescent microscopy images were taken using a Zeiss observer Z1 microscope. Flow cytometry analysis of LNP uptake in CEM cells was done by measuring fold increase in FL1 mean fluorescence intensity (MFI) using a flow cytometer (Beckman Coulter FC 500).

### Activation of latent HIV and primary CD4+ cells

J-Lat clone 8.4, clone 10.6 or peripheral blood mononuclear cells from healthy donors were incubated in RPMI + PS +10% FBS. Primary thymocytes were cultured in RPMI + PS +10% human AB serum. Flow cytometry analysis was performed on a Beckman Coulter FC 500 flow cytometer. For detection of latent HIV in a primary cell model, SCID-hu (Thy/Liv) implants were infected with 10 ng NL4-3 and the virus was allowed to spread for 4 weeks before tissue harvesting as described previously [37], [38], [39]. Single positive CD4 cells were isolated by negative selection using the CD4 negative isolation kit II (Miltenyi Biotec). KC57-RD1 antibody was purchased from Beckman Coulter and all other antibodies (CD69-PE-Cy7, CD4-PE, and CD8-APC) were purchased from BD Biosciences.

### NL4-3 spreading assay

Nelfinavir was incorporated into LNP in the same manner as bryostatin-2 (dissolved in the organic phase of lipids) and separated from free drug by PD10 columns. CEM cells were infected in bulk (600,000 cells in 200 ul volume) with 5 ng/ml NL4-3 in the presence of 10 µg/ml polybrene for 2 hr. Cells were washed and plated in the presence of each indicated condition at a concentration of 50,000 cells/well (1 ml total) in a 24-well plate. Culture supernatant was collected at 3 days post-infection for p24 protein analysis by ELISA.

### Ethics Statement

No human subjects were used in this study. Tissues from anonymous sources are not considered human subjects, and are thus not subject to Institutional Review Board review. Peripheral blood was obtained through the UCLA Blood Bank in an anonymous fashion. Similarly, fetal tissues were obtained in an anonymous fashion from Advanced Bioscience Resources, Alameda, CA.

This study was carried out in strict accordance with the recommendations in the Guide for the Care and Use of Laboratory Animals of the National Institutes of Health. The protocol was approved by the UCLA Animal Research Committee (approval #1996-058-43). All surgery was performed under ketamine/xylazine anesthesia, and all efforts were made to minimize suffering. For all survival surgeries carprofen analgesic was also employed.
